# Grb2 Is Regulated by Foxd3 and Has Roles in Preventing Accumulation and Aggregation of Mutant Huntingtin

**DOI:** 10.1371/journal.pone.0076792

**Published:** 2013-10-08

**Authors:** Shounak Baksi, Nihar R. Jana, Nitai Pada Bhattacharyya, Debashis Mukhopadhyay

**Affiliations:** 1 Structural Genomics Division, Saha Institute of Nuclear Physics, Kolkata, India; 2 Division of Cellular and Molecular Neuroscience, National Brain Research Centre, Manesar, Haryana, India; 3 Crystallography & Molecular Biology Division, Saha Institute of Nuclear Physics, Kolkata, India; National University of Singapore, Singapore

## Abstract

Growth factor receptor protein binding protein 2 (Grb2) is known to be associated with intracellular growth and proliferation related signaling cascades. Huntingtin (Htt), a ubiquitously expressed protein, when mutated, forms toxic intracellular aggregates - the hallmark of Huntington’s disease (HD). We observed an elevated expression of Grb2 in neuronal cells in animal and cell models of HD. Grb2 overexpression was predominantly regulated by the transcription factor Forkhead Box D3 (Foxd3). Exogenous expression of Grb2 also reduced aggregation of mutant Htt in Neuro2A cells. Grb2 is also known to interact with Htt, depending on epidermal growth factor receptor (EGFR) activation. Grb2- mutant Htt interaction in the contrary, took place in vesicular structures, independent of EGFR activation that eventually merged with autophagosomes and activated the autophagy machinery helping in autophagosome and lysosome fusion. Grb2, with its emerging dual role, holds promise for a survival mechanism for HD.

## Introduction

Several neurodegenerative diseases are caused by the increase in number of glutamine (polyQ) in specific genes, known as polyQ expansion diseases. Expansion of polyQ results in the formation of aggregates or insoluble inclusions. Huntington’s disease (HD), the most well studied amongst nine such neurodegenerative disorders, is caused by polyQ expansion in the protein huntingtin (Htt) [1]. In order to decipher the normal biological function of Htt, critical to the understanding of HD pathology, several groups have observed the localization of Htt in vesicles, ER and nuclei in various cell models [2-4] and the N-terminal 18 amino acids of Htt were reported to constitute the membrane targeting domain that mediated the association of Htt with ER and late endosomes [2].

Growth factor receptor protein binding protein 2 (Grb2) is known to be an interactor of Htt and this interaction is reported to be regulated by the activation of epidermal growth factor (EGF) receptors [5]. The SH3 domains of this adaptor protein bind to the proline-rich regions of the guanine nucleotide releasing factor son of sevenless (SOS-1) and upon growth factor receptor activation and tyrosyl phosphorylation, they bring SOS-1 in close proximity of membrane bound Ras, eventually activating Ras and the downstream mitogen activated protein kinase (MAPK) cascade [6]. Grb2 is also involved in Rab5 mediated receptor endocytosis [7], a major pathway of epidermal growth factor receptor (EGFR) internalization in many cells [8]. In neurons, Grb2 is thought to link intracellular signaling cascades and activated receptor tyrosine kinases, like Trk receptors, and regulate neural survival, development, function, and plasticity [9]. It is suggested that Grb2-SOS-1 and Grb2-Htt are two different signaling complexes and since both Htt and SOS-1 bind to SH3, Htt acts as a competitor of the Ras-dependent signaling pathway [5]. Reports from transgenic and knockout animal models, protein-protein-interaction studies and the discovery of a plethora of Htt interactors suggest that Htt might act as a multifunctional scaffold during the process of clathrin-mediated endocytosis, neuronal transport processes and post synaptic signaling [10].

In the present study, we have examined the cellular fate of Grb2-Htt interaction in the context of HD. We have shown that Grb2 is upregulated in both R6/2 mice and STHdhQ^111/111^ cell lines. While investigating the cause of Grb2 upregulation in HD model we found several transcription factor binding sites in the upstream DNA sequence of *Grb2* and, through rational selection, checked the regulation of *Grb2* by Forkhead Box D3 (Foxd3), a member of the forkhead box (Fox) family of transcription factors [11]. The Grb2 upregulation has been shown to be a consequence of overexpression of Foxd3. We have demonstrated how Grb2 could regulate the aggregation propensity of mutant Htt and the predominance of its interaction with mutant Htt in the absence of EGFR activation. Subsequent alterations in the downstream signaling pathways suggested the way by which Grb2 could be associated in clearing the toxic load of mutant Htt. The study clearly postulates an alternate possibility of prevention of aggregation and clearance of Htt in HD models.

## Materials and Methods

### Ethics Statement

All the animal related experiments were performed according to the protocol approved by the Institutional Animal Ethics Committee of National Brain Research Centre, Manesar. The animals had free access to pelleted diet and water *ad libitum*. All efforts were made to minimize animal suffering.

### Plasmid Constructs

Details of plasmids, whether received as kind gift or described previously are given in supplementary material (File S1). For luciferase assay we cloned the 5’ upstream promoter region of mouse Grb2 gene from position -105 to -372 (encompassing the Foxd3 binding site from position -226 to -237) in pGL3 basic vector (designated as Luc-Grb2ups) between the restriction sites Xho1 and HindIII. The primer sequences used for this cloning are given in table S1 in File S2.

### Bioinformatics Tools

Upstream sequence of the mouse Grb2 was downloaded from ENSEMBLE and NCBI and Transfac® MATCH1.0 public online search tool was used with default parameters to find out the binding sites for transcription factors in the given DNA sequence. We checked the expression levels of these hits obtained in the available microarray data for HD [12,13] using gene expression atlas.

### R6/2 Mice

Ovarian transplanted hemizygote females carrying HD exon 1 gene with about 150 CAG repeats (strain name: B6CBATg (Hdexon1)62Gpb/3J) were purchased from Jackson Laboratory and crossed with B6CBAF1/J males. The transgenic strain was maintained by crossing the carrier males with CBA females. The genotyping was carried out using PCR as described previously by Mangirani et al., 1996 [14]. Age matched transgenic and wild type mice were used for all experiments as in [15]. The transgenic mice along with their age-matched controls were anesthetized and then perfused with PBS containing 4% PFA in PBS; brain samples were collected followed by cryosectioning with 20 mm thickness.

### STHdhQ^7/7^ and STHdhQ^111/111^ cells

STHdhQ^7/7^ cells express full-length wild type HTT with 7Q (homozygous) while STHdhQ^111/111^ cells express full length mutated HTT with 111Q (homozygous) from the chromosomal region and is considered as models for HD. These cell lines were established from wild type and homozygous mutant Hdh knock in embryonic mice respectively [3]. Dr. Marcy E. MacDonald of Massachusetts General Hospital, USA, kindly gifted/donated these cells to us and they have published that STHdhQ^111/111^ cells exhibited dominant HD phenotypes and indicated a disruption of striatal cell homeostasis by the mutant HTT protein, via a mechanism that was different from its normal activity (STHdhQ^7/7^ cells) [3]. This cell model of HD has been extensively used for identifying molecular alterations in HD [13,16-21].

### Cell Culture and transfection

Neuro 2A cells were procured from National Cell Science Centre (Pune, India) with proper licensing and routinely grown in DMEM (HiMedia, India) supplemented with 10% fetal bovine serum (Biowest, USA) at 37°C in 5% CO2 atmosphere under humidified condition as mentioned earlier [22-24]. Immortalized striatal HD cell lines, STHdhQ^111/111^ and STHdhQ^7/7^ cells [3] were grown in DMEM (HiMedia, India) supplemented with 10% FBS and 400µg/ml G418 (Invitrogen, USA) at 33°C in humidified condition and 5% CO_2_. Transfection of cells was performed using Lipofectamine ^TM^2000 transfection reagent (Invitrogen, USA). In case of co-transfection, constructs were taken in equal proportions. After 48hrs, transiently transfected cells with (transfection efficiency varied between 70-90%) were checked for transfection efficiency by monitoring GFP or Dsred expression under fluorescence microscope and were used for experiments.

### Antibodies and Chemicals

Chemicals and antibodies used in this study are described in File S1.

### RNA preparation

Total RNA was prepared from cultured cells using TRIzol Reagent (Invitrogen, USA) according to manufacturer’s protocol. RNA samples were quantitated using nanodrop 2000 spectrophotometer (Thermo scientific, USA).

### RNA Isolation from Mouse Tissue Samples

RNA isolation from mouse tissue samples was described previously [25] and is described in File S1.

### Real Time PCR and Analysis

For real time PCR quantification of mRNAs 1 µg of total RNA was subjected to DNase (Sigma) treatment followed by cDNA preparation using random hexamer primer (Fermentas), dNTPs and MuLv- Reverse transcriptase (Fermentas) following the procedure described in [26]. Real time PCR was done in Applied Biosystems 7500 real time PCR system. Data show mRNA expression levels relative to those of beta-actin; the former was then normalized to control expression levels for each experiment.

### Protein Extraction and Western Blot Analysis

Protein extraction protocol for western blot experiments from cell lines is described in supplementary material (File S1). The proteins from mice were prepared as described previously [15].

### Immunoprecipitation Assay

Immunoprecipitation was done using anti-Grb2 or anti -Htt antibody from STHdhQ^7/7^ and STHdhQ^111/111^ cell extracts and anti–Grb2 antibody for Httex1 GFP transfected Neuro2A cell extracts. 50 µg of the whole cell extract was used as input in each experiment and 200 µg extract was incubated overnight with antibody and Protein A/G beads. After SDS–PAGE and western transfer, presence of Htt in the Grb2 interaction complex was revealed by immunoblotting with anti-Htt (CST, USA), anti–Grb2 (Abcam, USA) and anti-GFP (Roche, USA) antibodies respectively.

### Knockdown of Grb2 by siRNA

Knockdown of *Grb2* by gene specific siRNA in Neuro2A cells was described earlier [22]. Grb2 knockdown in STHdhQ^7/7^ and STHdhQ^111/111^ cells was done with the same siRNA. Grb2si and Grb2scrmbsi clones were transfected in STHdhQ^111/111^ cells using Lipofectamine 2000 (Invitrogen, USA) using a protocol provided by the manufacturer. Transfected cells were selected by hygromycin resistance. Knockdown of *Grb2* was confirmed by western blot using anti-Grb2 antibody.

### Confocal Microscopy

Imaging was performed on LSM 510 META confocal laser scanning microscope equipped with an argon-krypton laser (Carl Zeiss, Germany). Cells were grown on L-lysine coated cover slips, fixed with 4% paraformaldehyde and washed with PBS, mounted on slides and images were acquired in 63X oil immersion objective [27]. ImageJ software was used for the calculation of Pearson’s correlation coefficient and Intensity correlation quotient.

### Aggregate Counting

Neuro2A cells were grown on coverslips in 35mm culture dish (Nunc, USA) transfected with 145Q Httex1 GFP and also cotransfected with Grb2-Dsred and Dsred as control and incubated for 48hr at 37°C. After 48hr of transfection coverslips were washed with PBS and mounted on slides for aggregate counting using confocal microscope (Carl Zeiss LSM 510 Meta, Germany). An average of 500 cells was counted for each slide and the experiments were done ten times.

### Fluorescence Recovery after Photobleaching

FRAP assay was described previously [27].

### Immunocytochemistry

Immunocytochemistry protocol is elaborated in File S1.

### Fluorescence Lifetime Imaging Microscopy

Fluorescence Lifetime Imaging Microscopy (FLIM) was carried out using LSM510 META (Carl Zeiss) microscope and two photon Ti-Sapphire femtosecond pulsed laser Spectra Physics Mai-Tai at 940nm excitation wavelength with Becker & Hickl GmbH TCSPC FLIM module attachment (SPC-150 TCSPC module and DCC-100 detector) and images were analyzed using SPC Image 2.9.1 software.

### Lysosomal Inhibition

BafilomycinA1 (Sigma, USA) was used as a lysosomal inhibitor for the assay. Cells were treated with 100nM of BafilomycinA1 and incubated for 12 hr and harvested for western blot analysis. Levels of LC3 were checked using western blot, which were normalized using the same of beta-actin.

### Chromatin immunoprecipitation (ChIP) assay

Methods used for the ChIP experiments were described earlier [28] and also in File S1.

### Filter Retardation Assay

Filter retardation assay was performed as described previously [27]. In brief, cells were lysed using 50 mm Tris–HCl (pH 8.0), 100 mm NaCl, 5 mm MgCl_2_, 0.5% NP-40 and protease inhibitor cocktail. Insoluble material was pelleted by centrifugation for 10 min at 18,000*g* at 4°C and were resuspended in 100 µl DNase I buffer [20 mm Tris–HCl (pH 8.0) and 15 mm MgCl_2_], and DNase I (Sigma, USA) was added to a final concentration of 0.5 mg/ml followed by incubation at 37°C for 1hr. Protein concentration was determined and the samples were diluted into 100 ml of 1% SDS and 50 mm DTT in PBS, boiled for 5 min; and filtered through a PVDF membrane using a BRL Hybrislot manifold. Two washes were performed with 200 µl of 0.1% SDS and then processed for immunodetection in the similar way of a regular western blot with anti-poly Q antibody (Chemicon, USA).

### Luciferase Assay

The method for the luciferase assay was described previously [29].

### MTT assay

MTT assay protocol is described previously [30], and is also discussed in File S1.

### In-vivo Chaperone assay

Chaperone potential of Grb2 was tested by transfecting pGL3 basic vector into Neuro2A cells along with Grb2-Dsred, Hsp-70 GFP (positive control) or Dsred (negative control). 24hr. after transfection the cells were given heat-shock at 42°C for 1 hr followed by recovery at 37°C for 0hr, 2hr and 6hr respectively. Cells were lysed and luciferase assay was performed as described above. Increased luciferase activity suggests chaperone like potential of the transfected protein.

### Grb2 purification

Briefly, Grb2-pET28a construct was transformed into BL21 (DE3) *E. coli* strain and grown until it reached 0.6 OD and then IPTG was used for induction following which the medium containing cells were incubated at 37°C. The cells were pelleted, lysed and sonicated and then His-tagged protein was purified using Ni-NTA resin (Qiagen, USA) and immidazole, according to manufacturer’s protocol. Protein fractions were checked for expression by running SDS-PAGE and fractions containing only purified Grb2 were used for further experiments.

### In-vitro Chaperone Assay

Insulin aggregation assay was done as described previously [31], and is also mentioned in supplementary material (File S1).

### Statistical Analysis

For statistical analysis, an unpaired ‘t’ test was carried out to compare the means of two experimental groups using the online software Graph Pad QuickCals available at Http://www.graphpad.com/quickcals/ttest.cfm. Statistical significance is shown with asterisks: * p ≤ 0.05; ** p ≤ 0.01, *** p≤ 0.001

## Results

### Grb2 is upregulated in Huntington’s disease Models

**Figure 1:**
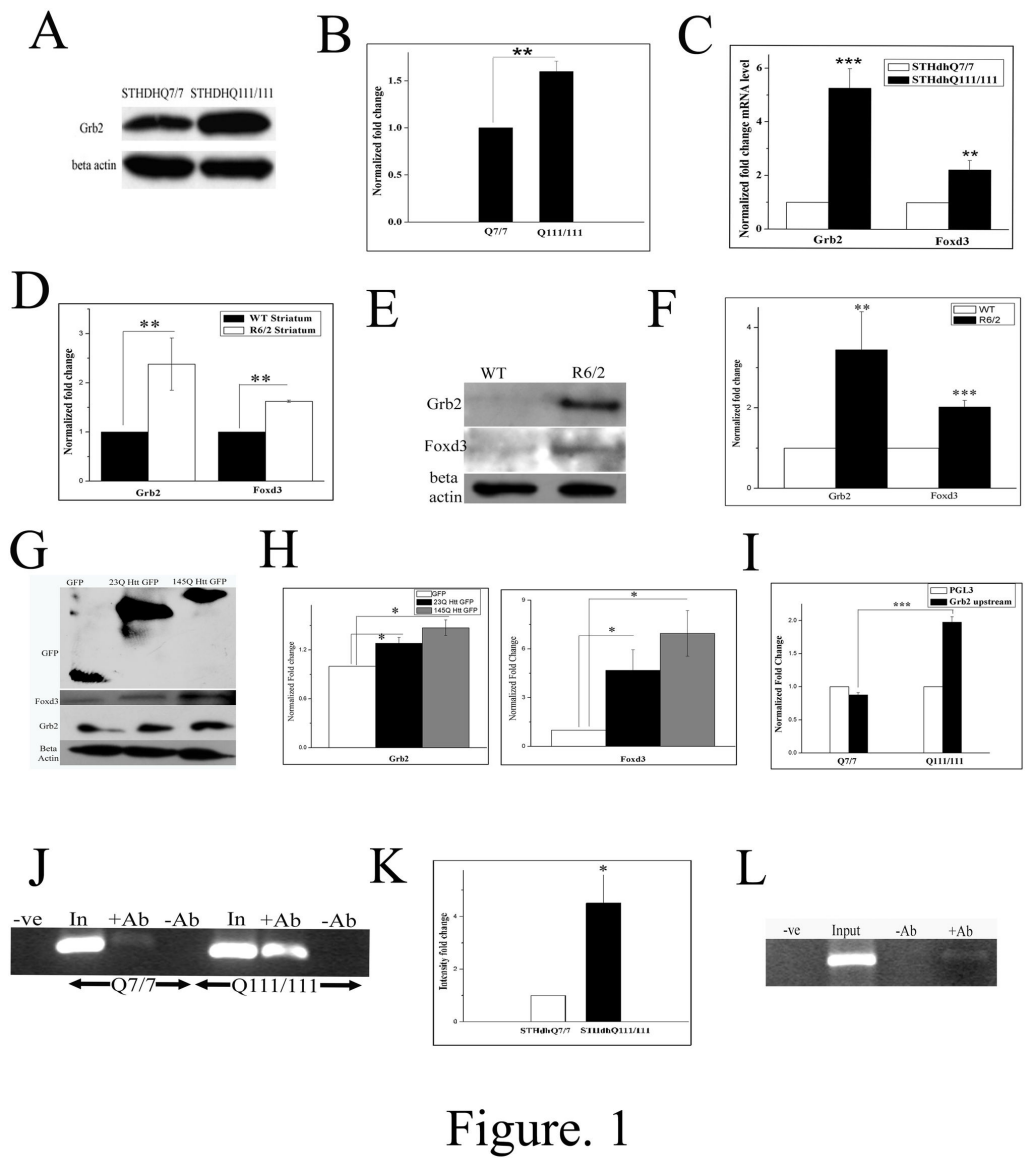
Grb2 is upregulated and is regulated by Foxd3 in Huntington’s disease condition A. Representative Western blot of three (n=3) independent experiments for Grb2 and beta actin expression in STHdhQ^7/7^ and STHdhQ^111/111^ cells. Expression of beta actin was taken as internal control. B. Bar diagram representing the mean optical density of bands obtained in A normalized to that of beta actin (p< 0.01). C. Normalized fold changes of Grb2 and Foxd3 mRNA levels of three independent real time PCR experiments in STHdhQ^111/111^ with respect to STHdhQ^7/7^, beta actin taken as internal control (n=3, p<0.001, and p<0.01 respectively). D. Grb2 and Foxd3 normalized fold change mRNA expression levels in striatum tissues of wild type and R6/2 age matched mice, beta actin taken as internal control. Data of three independent experiments (Grb2; p<0.01; Foxd3 p<0.01, n=3). E. Western blot showing Grb2, Foxd3 and beta actin levels in striatum tissues of age matched control and R6/2 mice F. Bar diagram of four (n=4; Grb2 p<0.01; Foxd3 p<0.001) independent samples as shown in E. G. Western blot representative picture of GFP, Grb2, Foxd3 and beta actin (loading control) expression levels in Neuro2A cells transfected with GFP, 23Q Httex1 and 145QHttex1 GFP respectively. H. Bar diagram of expression levels of Grb2 and Foxd3 normalized to that of beta actin as shown in panel H. of three independent experiments (p<0.05,n=3). I. Luciferase reporter assay (n=3) of the Grb2 upstream sequence cloned in pGL3 vector in empty GFP transfected STHdhQ^7/7^ and GFP transfected STHdhQ^111/111^ cells. Luciferase activity of above cells was normalized by the luciferase activity of the corresponding empty pGL3 vector transfected cells (p<0.001). J. Comparative ChIP analysis showing relative occupancy of endogenous Foxd3 in Grb2 promoter was more in STHdhQ^111/111^ cells compared to STHdhQ^7/7^ cells. Immunoprecipitation was carried out with anti-Foxd3 antibody and the immunoprecipitated DNA was PCR amplified using primers specific for Grb2 upstream sequence (n=3). K. Bar diagram of normalized fold change of intensity of immunoprecipitated DNA as shown in panel J. Data of three independent experiment (n=3, p<0.05). L. Representative agarose gel picture of ChIP analysis (n=3) showing occupancy of endogenous Foxd3 in Grb2 promoter in Neuro2A cell.

We checked the levels of endogenous Grb2 in STHdhQ^111/111^ and STHdhQ^7/7^ cells by western blot and the relative levels of endogenous Grb2 was found to be upregulated by about 1.5 folds in STHdhQ^111/111^ cells with respect to that of STHdhQ^7 /7^cells (Figure 1A,B p<0.01, n=3). Also mRNA levels of Grb2 was found to be upregulated in STHdhQ^111/111^ cells by about 5 fold (Figure 1C, p<0.001, n=3). We checked the levels of Grb2 mRNA and protein in age matched R6/2 mouse model of Huntington’s disease. R6/2 mice showed about 2.5 fold (Figure 1D; n=3, p<0.01) upregulation in mRNA level as well as 3 fold upregulation in protein level (Figure 1E,F, n=4, p<0.01) in striatum with respect to wild type mice. We transfected Httexon1GFP constructs with two polyQ lengths (23Q and 145Q) in Neuro2A cells and found Grb2 to be upregulated as a result of Httexon1 transfection (Figure 1 G,H; n=3, p<0.05). Although the differential expression of Grb2 between 23Q and 145Q Httexon1 transfected cells cannot be taken as significant (p=0.0506; n=3), despite a consistent transfection efficiency otherwise, the intensity of GFP in Htt145Qex1 transfected cells was less compared to that in Htt23Qex1 transfected cells nonetheless.

### Foxd3 predominantly regulates the transcription of Grb2

While investigating for molecular players behind Grb2 transcriptional upregulation, we used Transfac® MATCH1.0 public online search tool for probable binding sites of transcription factors in the 10Kb upstream DNA sequence of *Grb2* and found 30 probable binding sites for 19 different transcription factors (see Tables S2 and S3 in File S2). Of these only 6 were known to be differentially expressed in HD [12,13] and only 3 have binding sites within 1Kb DNA sequence upstream to *Grb2*. Combining these two selection criteria, only Foxd3 and HNF-3beta/Foxa2 came out to be two probable candidates for the regulation, of which Foxd3was chosen for our study. In STHdhQ^111/111^ cells the expression of Foxd3, member of forkhead box transcription factor, having a binding site at -226 to -237 upstream of *Grb2*, was found to be upregulated by 2 folds (Figure 1C, n=3; p<0.01). In age matched R6/2 mice Foxd3 was upregulated in striatum by 1.5 fold (Figure 1D, n=3, p<0.01) at the mRNA level and 2 fold at the protein (Figure 1E, F, n=4, p<0.001) level. Consequently Foxd3 was also upregulated when Neuro2A cells were transfected with Htt exon1 (23Q, 145Q) GFP (Figure 1 G, H; n=3, p<0.05). Here also the differential expression of Foxd3 between 23Q and 145Q Httexon1 transfected cells (p=0.1056; n=3) was not significant, despite a consistent transfection efficiency otherwise, and the intensity of GFP in 145QHttexon1 transfected cells was diminished compared to the other.

Luc-Grb2ups Foxd3 binding site was transfected both in STHdhQ^7/7^ and STHdhQ^111/111^ cells and it showed 2 fold enhanced luciferase signal in case of the latter, indicating that the promoter activation by Foxd3 was higher in the latter cell type due to the upregulation of Foxd3 (Figure 1I, p<0.001, n=3). Chromatin immunoprecipitation (ChIP) assay was then used to validate actual binding of Foxd3 to the upstream nucleotide sequence of the gene *Grb2*. We immunoprecipitated the crosslinked nuclear extract from both the cells with anti-Foxd3 antibody and did PCR with Grb2ups specific primers. The intensity of DNA bands in agarose gel electrophoresis (Figure 1 J, K), indicated about 4 fold higher (n=3; p<0.05) immunoprecipitation in case of STHdhQ^111/111^ cells. Similar positive bands were observed in ChIP with Neuro2A nuclear fraction (Figure 1L, n=3).

### Upregulated Grb2 interacts with Htt in a polyQ dependent manner and deviates from its normal function

**Figure 2:**
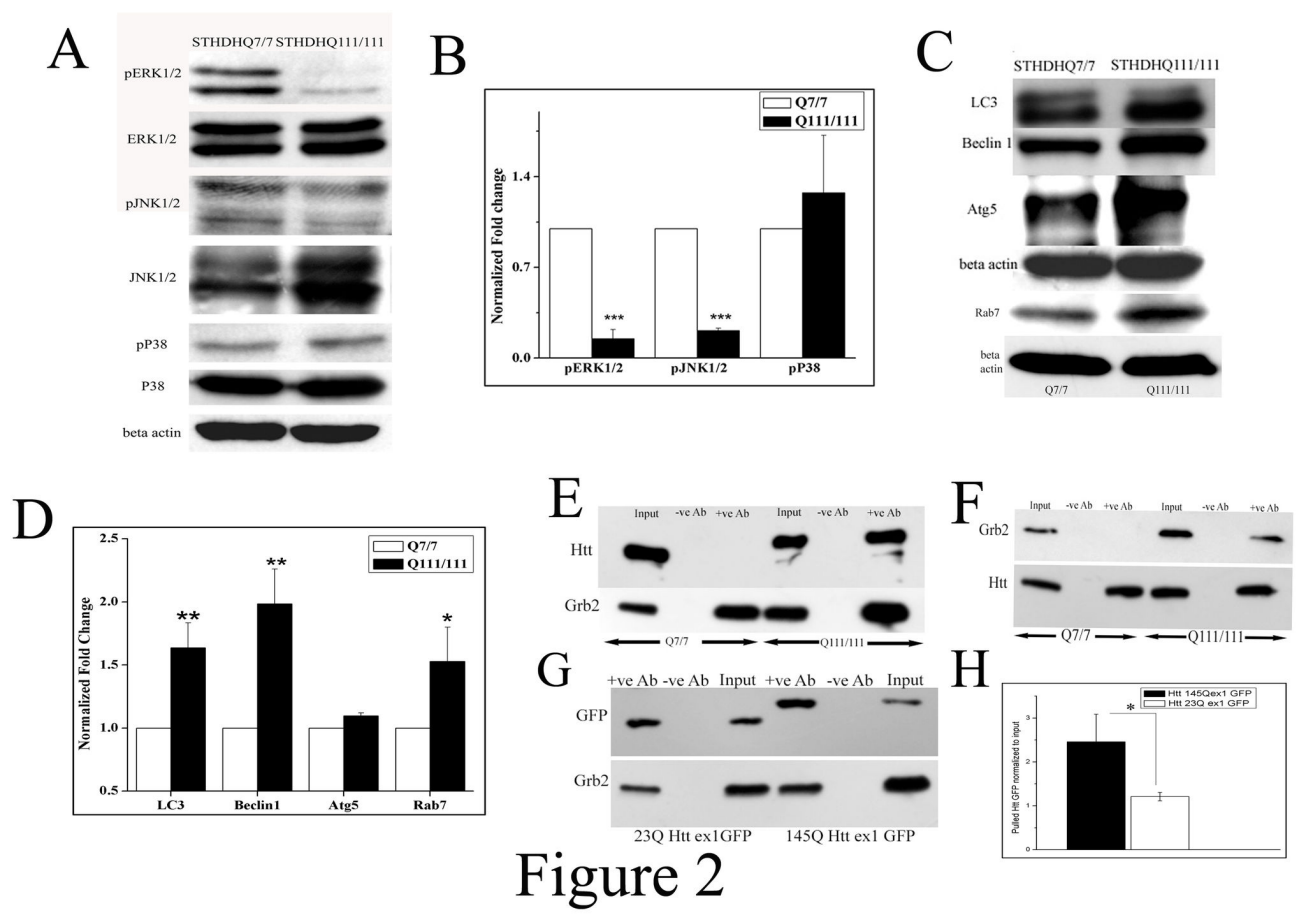
Upregulated Grb2 interacts with mutant Htt and deviates from its normal function A. Western blot analysis for the expression of phospho ERK1/2, ERK1/2, phospho JNK1/2, JNK1/2, phospho p38, p38 and beta actin levels in STHdhQ^7/7^ and STHdhQ^111/111^ cells. B. Bar diagram of densitometric analysis for three independent (n=3) experiments as shown in panel A the values of phospho ERK1/2 was normalized to that of total ERK1/2 levels, phospho JNK1/2 was normalized to total JNK and phospho p38 level was normalized to that of total p38 level (for pERK1/2 and pJNK1/2 ;p<0.001). C. Western blot analysis for the expression of LC3, beclin1, Atg5, Rab7 and beta actin in STHdhQ^7/7^ and STHdhQ^111/111^ cells. D. Bar diagram of densitometric analysis of three (n=3) different experiments as shown in panel C, beta actin taken as internal control (p<0.01 for LC3 and Beclin1, p<0.05 for Rab7). E. Immunoprecipitation experiment (n=3) of Grb2 with Htt in STHdhQ^7/7^ and STHdhQ^111/111^ cells. Cell extract was pulled with anti-Grb2 antibody and the pulled down protein was probed with Htt antibody in 6% SDS-PAGE and for the pulled Grb2 was run in 12% SDS-PAGE with same sample and probed with anti Grb2 antibody. F. Immunoprecipitation experiment (n=3) of Grb2 with Htt in STHdhQ^7/7^ and STHdhQ^111/111^ cells. Cell extract was pulled with anti-Htt antibody and the pulled down protein was probed with anti Grb2 antibody in 12% SDS-PAGE and for the pulled Htt was run in 6% SDS-PAGE with same sample and probed with anti Htt antibody G. Immunoprecipitation experiment (n=3) with Neuro2A cells transfected with 23Q and 145Q Httex1 GFP respectively and pulled with anti-Grb2 antibody, probed with anti GFP antibody. H. Bar diagram of densitometric analysis of three independent (n=3) as shown in panel G.

To ascertain the effects of enhanced level of Grb2 in STHdhQ^111/111^, under the transcriptional regulation of Foxd3, downstream signaling pathways of Grb2 were investigated. The activity levels of MAPK signaling molecules like ERK1/2 and JNK1/2 were assayed in terms of their extent of phosphorylation. Interestingly, phospho ERK1/2 (Figure 2A, B; p<0.001, n=3) and phospho JNK1/2 (Figure 2A, B p<0.001; n=3) levels were found to be downregulated significantly despite the upregulation of Grb2. In order to find out where this extra Grb2 was going, we investigated the levels of autophagy related proteins in STHdhQ^111/111^ cells and the two autophagy markers LC3, Beclin1 and late endosomal marker Rab7 were found to be significantly upregulated in the HD cell model (Figure 2C,D; p<0.01; n=3; for all).

The interaction between Grb2 and Htt was first confirmed by immunoprecipitation in both STHdhQ^7/7^ and STHdhQ^111/111^ cells. We pulled down the whole cell extracts by anti-Grb2 antibody and probed with anti-Htt antibody. We found that in STHdhQ^111/111^ cells Htt was indeed pulled down by Grb2 but not in STHdhQ^7/7^ indicating that the interaction between Grb2 and Htt was polyQ dependent (Figure 2 E, n=3). We confirmed this data by reverse pull down by anti-Htt-antibody and probing with anti-Grb2-antibody and we observed Grb2 being pulled down in case of STHdhQ^111/111^ but not in STHdhQ^7/7^ (Figure 2F, n=3) as expected. Since full length Htt is of very high molecular weight (around 350 kDa) and Grb2 being around 27 kDa it was impossible to probe both the molecules in the same gel. So Htt was probed in 6% SDS-PAGE and Grb2 in 12% SDS-PAGE with equal amounts of same samples. We also transfected Neuro2A cells with 23Q and 145Q Httexon1 GFP and immunoprecipitated with anti-Grb2 antibody and probed the immunoblot with anti-GFP antibody. We found about 2 fold higher precipitation in case of the mutant 145Q Httex1 GFP (Figure 2G, H, n=3, p<0.05). Clearly in the HD cell model, Grb2 has been digressed from its natural signal transduction role.

### Elevated level of Grb2 reduces Htt Aggregation and shows chaperone like activity

**Figure 3:**
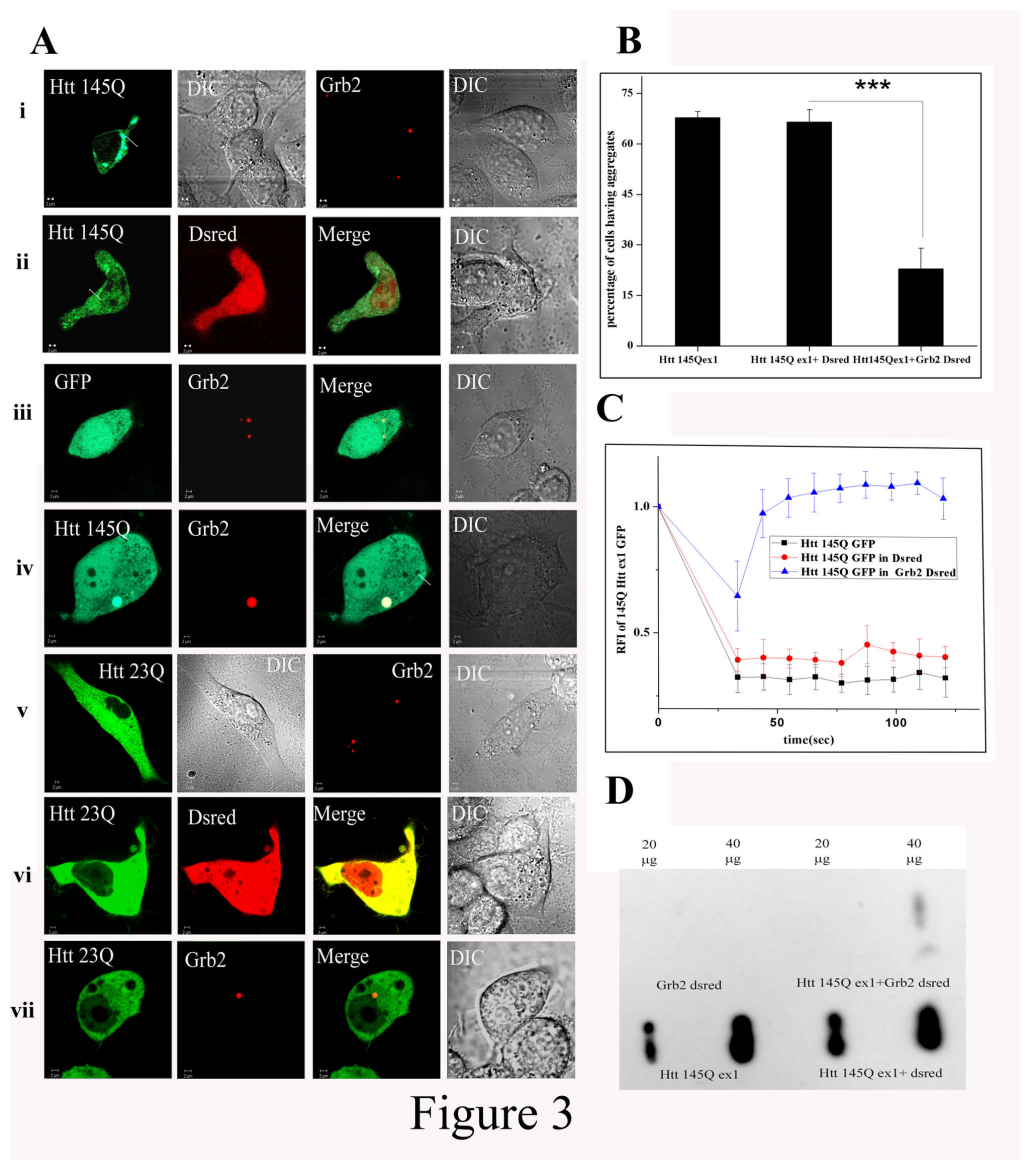
Grb2 reduces Htt exon 1 aggregates in Neuro2A cell A. Representative confocal images of Neuro2A cells transfected with i. 145QHttex1 GFP, i.Grb2-Dsred (Grb2 cloned in DsredC1 vector), ii. Double transfection with Htt 145QHttex1GFP and DsredC1 empty vector, iii. Double transfection of GFP empty vector and Grb2-Dsred, iv. Double transfection of 145Q Httex1 GFP and Grb2-Dsred, v. 23QHttex1 GFP and Grb2-Dsred again, vi.double transfection of 23QHttex1 GFP and DsredC1 empty vector and vii. Double transfection of 23QHttex1 GFP with Grb2-Dsred. All images were taken in same magnification. B. Bar diagram of percentage of Neuro2A cells having aggregates transfected with 145Q Httex1 GFP, co-transfection of 145QHttex1 GFP and empty vector DsredC1 and reduced percentage of cells with aggregates in co-transfection of 145QHttex1 GFP with Grb2-Dsred (n=10, p<0.001). C. Relative fluorescence index (RFI) of 145QHttex1 GFP pre- and post bleaching in cells transfected with 145QHttex1 GFP, co-transfected with 145Q Httex1 GFP and Dsred empty vector and cells co-transfected with 145Q Httex1 GFP with Grb2-Dsred. D. Representative image for filter retardation assay with Neuro2A cells transfected with Grb2-Dsred, 145Q Httex1 GFP double transfected with 145Q Httex1 GFP with Grb2-Dsred and double transfected with 145QHtt ex 1 GFP with Dsred. For all the samples input loads of 20µg and 40µg were used.

We studied the interaction of Htt exon1-Grb2 and its consequences in Neuro2A cells by confocal imaging. Two different forms of GFP fused Htt exon1 clones [23Q Htt exon1 GFP (23QHttex1GFP) and 145Q Htt exon1 GFP (145QHttex1GFP) respectively] were used with different polyQ repeats in the constructs. Full length Grb2 was expressed as a fusion product with red fluorescent protein Dsred (Grb2-Dsred) (Figure 3A). 23QHttex1GFP, when expressed alone, was found to be uniformly distributed in the cytoplasm, whereas 145QHttex1GFP, when expressed alone, was found to form aggregates inside the cells in addition to its ubiquitous expression throughout (Fig 3Ai.). Grb2-Dsred when expressed alone was localized in spherical structures (Fig. 3Ai), previously identified by us as late endosomal vesicles [22]. Surprisingly, we found that double transfected Neuro2A cells expressing both Grb2-Dsred and 145QHttex1GFP did not form any visible aggregates. In Neuro2A cells 145QHttex1GFP was also localized in vesicular structures (Fig.3Aiv) and found to colocalize with Rab7 (Figure S1 in File S2), a late-endosomal marker, along with Grb2 in addition to its ubiquitous expression in the cytosol. The average intensity of the GFP signal in vesicles was significantly higher than that in the cytoplasm and the protein was found to be colocalized with Grb2-Dsred inside the vesicles. The number of cells having aggregates was found to be significantly reduced in the double transfected Htt-Grb2 cells with respect to single transfected cells expressing 145QHttex1GFP (p<0.001; n=10; Figure 3B). In case of 23QHttex1GFP and Grb2-Dsred double transfected cells no such co-localization between Htt and Grb2 was observed (Fig. 3Avii).

**Figure 4:**
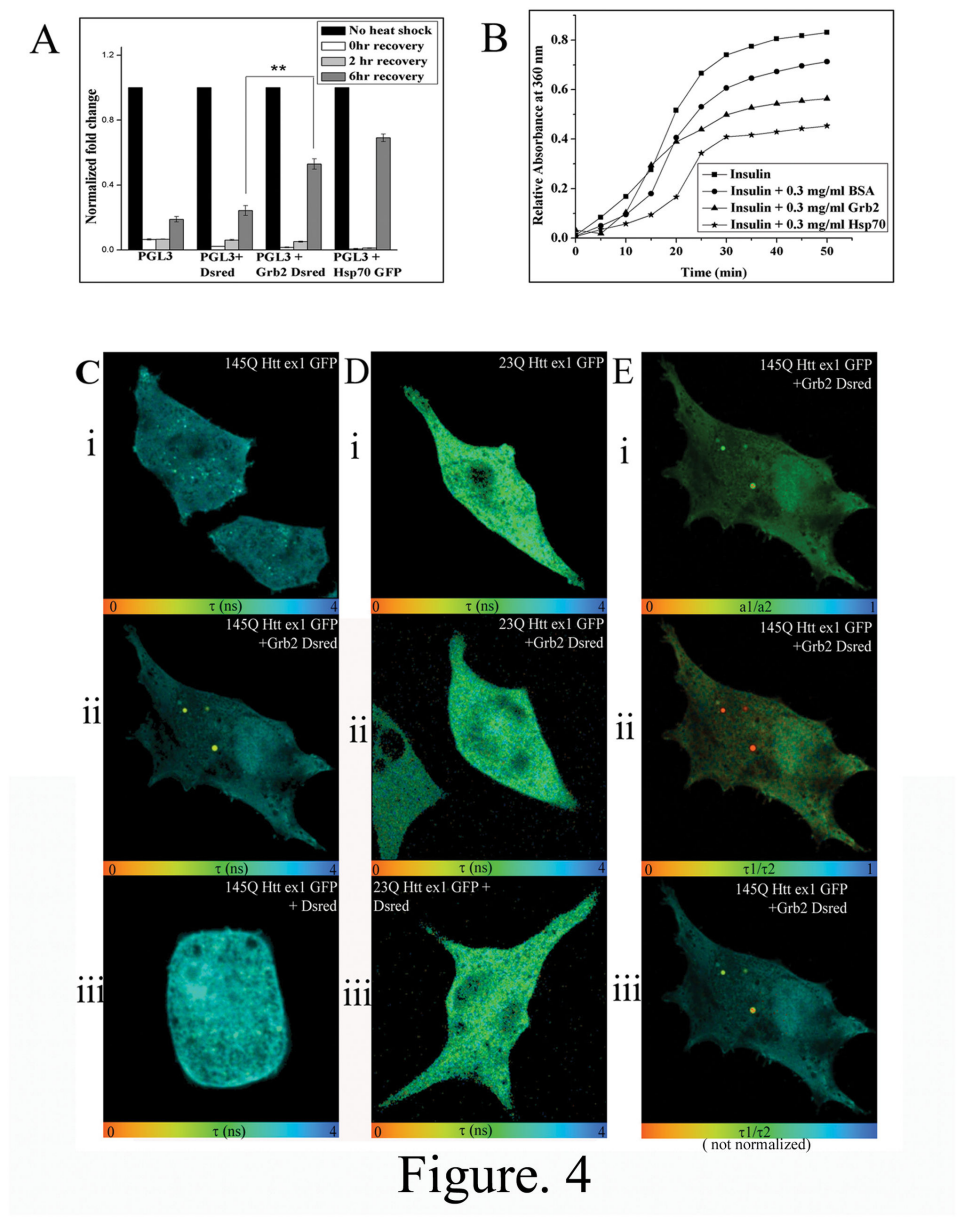
Localized interaction between Htt and Grb2 and Chaperone potential of Grb2 A. Normalized fold change of luciferase signal of cells transfected with pGL3 basic vector, pGL3 vector plus Dsred empty vector, pGL3 basic vector plus Grb2-Dsred and pGL3 vector plus Hsp70 GFP, all of the cells given heat shock for 1 hour and recovery at 37°C for 0hr, 2hr and 6hr. Fold change was calculated taking no heat shock cells as a control. B. Change in relative absorbance at 360nm with time was plotted for insulin with DTT alone or, in presence of 0.3mg/ml BSA, 0.3mg/ml Grb2, and 0.3mg/ml Hsp70. C. Fluorescence lifetime images of 145Q Httex1 GFP in cells transfected with i. 145Q Httex1 GFP, ii. co-transfected with 145Q Httex1 GFP and Grb2-Dsred and iii.co-transfected with 145Q Httex1 GFP and DsredC1. D. Fluorescence lifetime images of 23Q Httex1 GFP in cells transfected with i. 23Q Httex1 GFP, ii. Co-transfected with 23Q Httex1 GFP and Grb2-Dsred C and iii. co-transfected with 23Q Httex1 GFP and DsredC1. E. Analysis of cells co-transfected with 145Q Httex1 GFP and Grb2-Dsred i. a1/a2 image shows the ratio of interacting and non interacting species within the cell, ii and iii (normalized and not normalized images respectively) shows τ1/τ2 images in different scales showing the ratio of GFP two lifetime species within the cell.

Grb2-Dsred and 145QHttex1-GFP containing structures, despite having colocalization with Rab7, did not have resemblance with classical late endosomal vesicles. Grb2 was not any aggregate prone protein to our knowledge. To verify whether these structures were protein inclusion bodies or actually vesicles, we did Fluorescence recovery after photobleaching (FRAP) and filter retardation assay. Fluorescence recovery after photobleaching (FRAP) experiment was performed to investigate the mobility of the mutant Htt (145QHttex1GFP), in this case the one with the bigger polyQ stretch. Htt aggregates in single transfected cells with 145QHttex1 GFP, when bleached, failed to recover their fluorescence. But in cells co-transfected with 145QHtt GFP and Grb2-Dsred, full recovery of 145QHttex1GFP signals was observed after photobleaching after bleaching at vesicular structures (Figure 3C). Additionally, filter retardation assay showed no SDS insoluble aggregates or inclusion bodies in Grb2-Dsred transfected Neuro2A cells at both 20µg and 40µg protein loads but 145QHttex1 GFP transfected and 145QHttex1 GFP and Dsred double transfected cells showed visible aggregates in both 20 µg and 40 µg loads. In 145QHttex1 GFP and Grb2-Dsred transfected cells the band was merely visible at 40 µg input and no band was visible at 20 µg protein input (Figure 3D and Figure S2 in File S2). The data unequivocally indicated increased protein mobility of 145QHttex1 GFP in presence of enhanced proportions of Grb2-Dsred, suggesting a quantitative reduction of Htt aggregates in presence of the latter. The natural conclusion from these observations, that Grb2 could possess chaperone like potential, was checked by *in-vivo* and *in-vitro* chaperone assays. Grb2-Dsred showed significantly higher (Figure 4A, p<0.01, n=3) recovery with respect to cells transfected with only pGL3 or pGL3 and Dsred. Hsp70-GFP was taken as a positive control for this experiment.

Insulin aggregation assay, a well known assay for assessing chaperone action of a protein [31] was used to check the chaperone like activity of purified Grb2. Mutant Htt, being aggregation prone, could not be purified *in-vitro*. *In-vitro* insulin aggregation was induced by adding DTT to it and light scattering at 360nm was monitored for 50 mins. In presence of purified His-tagged Grb2 the scattering for insulin aggregation was lower than that in presence of BSA but it was higher than that of Hsp70 (Figure 4B).

### Interaction between mutant Htt and Grb2 takes place within vesicles

Given that Grb2 directly influenced the aggregation state of Htt, we wanted to know exactly where did they cross-talk and time correlated single photon counting (TCSPC) based Fluorescence lifetime imaging (FLIM) techniques were used to check Förster resonance energy transfer (FRET) between the two molecules in Neuro2A cells. Two photon excitation femtosecond pulsed laser was used to detect GFP lifetime. The clones used were standard FRET donor-acceptor pair (GFP donor and Dsred acceptor). The lifetime map of double transfected cells revealed strong reduction of GFP lifetime in vesicular bodies (Figure S1 in File S2) inside cells (Figure 4C panel ii) indicating proximity of the molecules within Förster distance. No such reduction in GFP lifetime was observed in control experiments with empty vectors (cells co-transfected with 145QHttex1 GFP and Dsred) (Figure 4C panel i and iii). The distribution of the ratio of interacting and non interacting components of 145QHttex1 GFP in the cell (a1/a2) and the ratio of the two GFP species having different lifetimes across the cell (τ1/τ2) are shown in Figure 4E(i and ii, iii) respectively. The lifetime image of 23QHttex1 GFP co-transfected with Grb2-Dsred did not show any such reduction in GFP lifetime (Figure 4D, ii). The results strongly depicted that Htt-Grb2 interaction was being carried out in vesicular structures (Figure S1 in File S2) inside cells.

### Grb2 helps in Htt clearance by evoking lyso-autophagy pathway and elicits autophagosome-lysosome fusion

**Figure 5:**
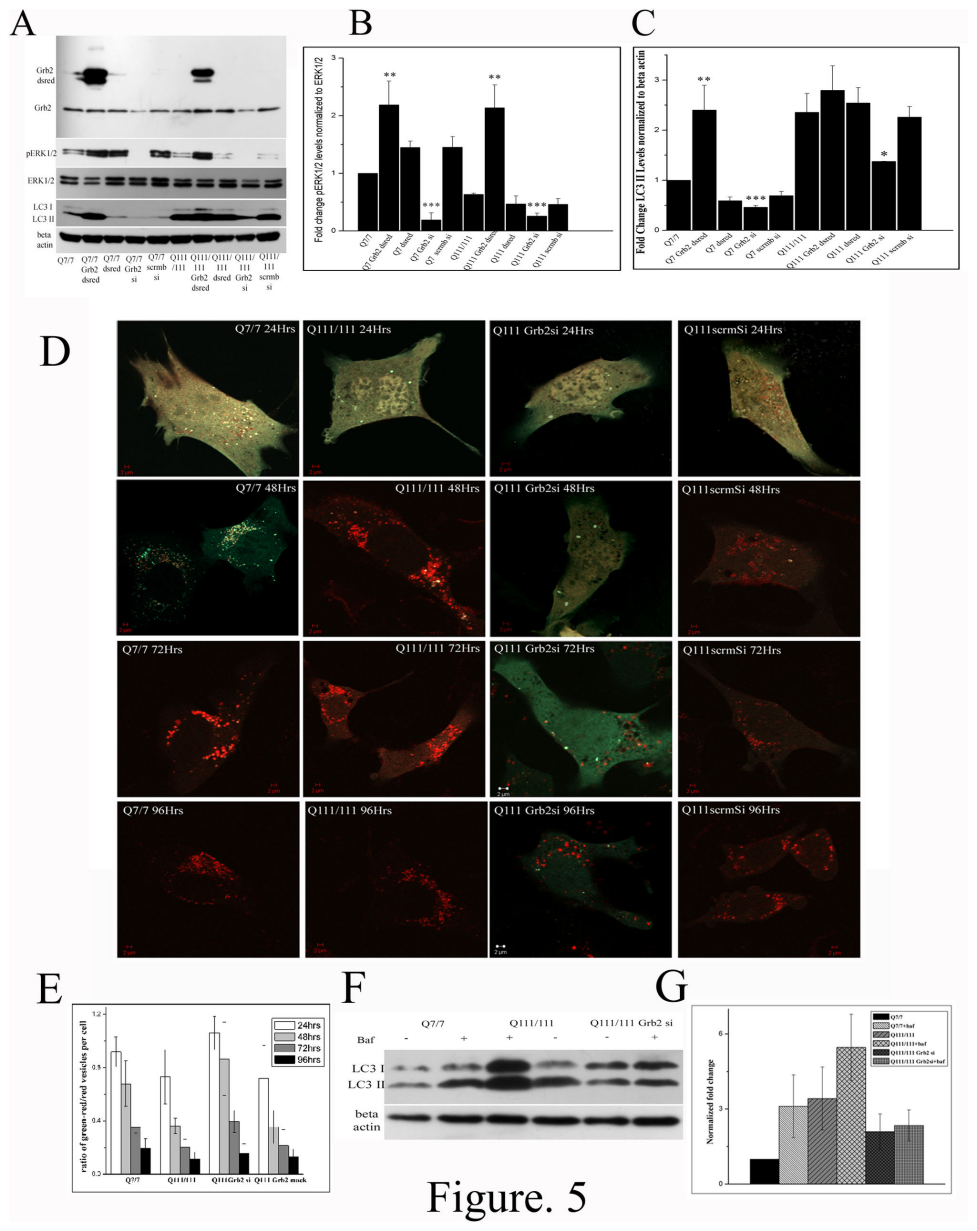
Grb2 helps in Htt clearance via lyso-autophagy pathway and also in autophagosome fusion with Lysosome A. Western blot analysis of expression of Grb2, pERK1/2, total ERK, LC3 and beta actin in STHdhQ^7/7^, STHdhQ^7/7^ transfected with Grb2-Dsred, STHdhQ^7/7^ transfected with Dsred, STHdhQ^7/7^ Grb2si, STHdhQ^7/7^ Scrmbsi, STHdhQ^111/111^ transfected with Grb2-Dsred, STHdhQ^111/111^ transfected with Dsred, STHdhQ^111 /111^Grb2si and STHdhQ^111/111^ Scrmbsi cells. B. Bar diagram of pERK1/2 levels of three experiments (n=3) as shown in panel A. normalized to total ERK1/2 levels. C. Bar diagram of LC3 II levels of three experiments (n=3) as shown in panel A normalized to beta actin levels. D. Representative confocal images of STHdhQ^7/7^, STHdhQ^111/111^, STHdhQ^111 /111^Grb2si, STHdhQ^111/111^ Scrmbsi cells transfected with dual tagged GFP-LC3-mCherry at 24hrs, 48hrs, 72hrs and 96hrs timepoints. E. Bar diagram of ratio of number of green/red to free red vesicles in each cell of D (n≥20). F. Representative western blot showing LC3 and beta actin levels of STHdhQ^7/7^, STHdhQ^111/111^ and STHdhQ^111 /111^Grb2si cells treated or untreated with 100nM bafilomycinA1, G. Bar diagram of three independent (n=3) experiments of F. of LC3II levels normalized to beta actin levels.

To understand any correlation between the vesicular localization of Htt-Grb2 interaction and consequent rise in the levels of autophagy markers (also previously reported in some HD models [32]), Grb2 was knocked down in both STHdhQ^7/7^ and STHdhQ^111/111^ cells using RNAi molecules, which showed downregulation of LC3 (Figure 5A, C, p<0.001 and p<0.05, n=3 respectively) and pERK1/2 (Figure 5A, B p<0.001 for both, n=3). When the same was overexpressed in STHdhQ^7/7^ and STHdhQ^111/111^ cells by Grb2-Dsred transfection, expression of pERK1/2 was upregulated (Figure 5A, B, p<0.01 for both, n=3) and that of LC3 was marginally upregulated (Figure 5A, C p<0.01 for STHdhQ^7/7^, n=3).

We also checked the temporal distribution of LC3 in lysosomal vesicles using a LC3-GFP-mCherry construct which was supposed to emit only free red signals on merger with lysosomes. STHdhQ^7/7^ cells, up to 48hrs timepoint, showed predominantly green signals that turned red after 72hrs. STHdhQ^111/111^ cells, on the other hand, showed green/red to free red transition of signal within 48hrs. In STHdhQ^111/111^ Grb2si cells the lysosomal merger took place very late and only at 96hrs the vesicles became red (GFP quenched). The STHdhQ^111/111^ scrmbsi cells showed similar results as STHdhQ^111/111^ cells (Fig: 5D, E). This result showed that STHdhQ^111/111^ Grb2si cells were not efficient in lysosomal fusion as compared to the STHdhQ^111/111^ cells, suggests that Grb2 helps in autosomal-lysosomal fusion. We also confirmed this observation by treating the cells with a well known lysosomal inhibitor BafilomycinA1. BafilomycinA1 treatment showed increased LC3II levels in both STHdhQ^7/7^ and STHdhQ^111 /111^cells (Figure 5 F, G; n=3) due to inhibition in lysosomal fusion. But in STHdhQ^111/111^ Grb2si cells no significant change in LC3II levels (Figure 5 F, G; n=3) were observed after bafilomycinA1 treatment, indicating inability of these cells in lysosomal fusion with autophagosomes.

We checked the survivability of the STHdhQ^7/7^, STHdhQ^111/111^ and STHdhQ^111/111^ Grb2si cells using MTT assay (Figure S3 in File S2). The STHdhQ^7/7^ cells showed maximum survivability followed by STHdhQ^111/111^ cells and the Grb2si cells showed least survivability amongst the three, possibly due to some effects of knock-down mechanism of Grb2, which also induced growth retardation. Grb2 transfection increased the survivability in all three cases (Figure S3 in File S2). The Dsred transfected cells showed lower survivability compared to normal cells in all cases, this was possibly due to Lipofectamine induced stress in the cells.

### Endogenous mutant Htt and Grb2 colocalize with autophagosome

**Figure 6:**
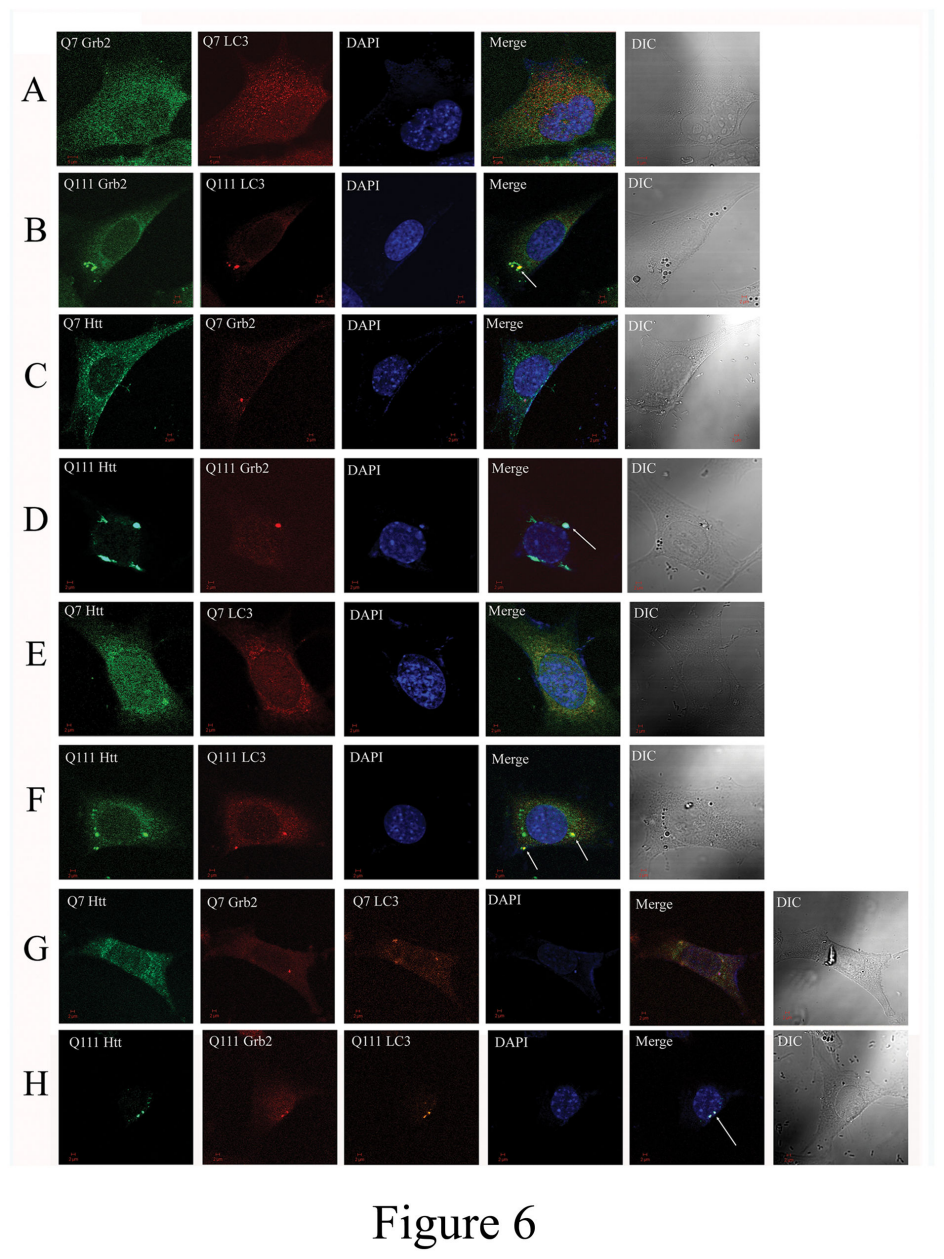
Endogenous Htt and Grb2 is localized to Autophagosome in STHdhQ^111/111^ cell Double stained confocal images of Htt, Grb2 and LC3 in STHdhQ^7/7^ and STHdhQ^111/111^ cells. A & B. Grb2 & LC3 in, C & D Htt & Grb2, E&F. Htt & LC3 G&H triple stained images in STHdhQ^7/7^ and STHdhQ^111/111^ cells respectively.

To ascertain the nature of the vesicles where endogenous Htt and Grb2 were colocalized, we stained STHdhQ^7/7^ and STHdhQ^111/111^ with specific monoclonal antibodies against the proteins and secondary antibodies tagged with alexa 488 or alexa546, respectively. The single stained cells with anti-Htt antibody showed thread like structures in STHdhQ^111/111^ but in STHdhQ^7/7^ cells it showed punctate distribution spread over the cells (Figure S4 in File S2). When stained with anti-Grb2 antibody alone, both the cells showed cytoplasmic distribution with much intense vesicular structures (Figure S4 in File S2). Simultaneous staining of both Htt and Grb2 showed colocalization of the two in STHdhQ^111/111^ cells but no such colocalization could be observed in STHdhQ^7/7^ cells (Figure 6 C, D ; ICQ and Pearson’s correlation coefficient values were significantly higher, p<0.01, n=20, Figure S5 in File S2). Further, taking a cue from western blot results, the cells were stained with the autophagosome marker anti-LC3 antibody. Both Htt and Grb2 were separately found to be colocalized with LC3 in STHdhQ^111/111^ cells but none of them were found to get colocalized with LC3 in STHdhQ^7/7^ cells (ICQ and Pearson’s correlation coefficient values were significantly higher, for Grb2 and LC3, Figure 6 A, B ICQ p<0.05 and Pearson’s correlation coefficient p<0.001, n=20 and for Htt and LC3, Figure 6 E, F Pearson’s correlation coefficient p<0.01, n=20; see also Figure S5 in File S2). The Htt-Grb2 colocalized structures were also found to be in colocalization with LC3 in STHdhQ^111/111^ cells in triple protein stained cells (Figure 6 H).

We noticed changes in endogenous Htt distribution in different conditions. In STHdhQ^7/7^ cells Htt had punctate distribution throughout the cell whereas in STHdhQ^111/111^ cells some fibril like structures were also observed in addition to the puncta. Surprisingly in STHdhQ^111 /111^Grb2si cells these fibrillar structures were not observed (Figure S6 in File S2). When Grb2 was overexpressed by transfection with Grb2-Dsred, STHdhQ^7/7^ showed no co-localization with Grb2-Dsred vesicles, whereas STHdhQ^111/111^ showed co-localization of endogenous Htt with Grb2-Dsred vesicle (Figure S5 in File S2). In case of STHdhQ^111 /111^Grb2si cells also this co-localization was observed (Figure S6 in File S2).

## Discussion

In the present study, the cellular fate of Grb2 Htt interaction is checked. For the first time, it is demonstrated that endogenous Grb2 is upregulated in HD R6/2 mouse model as well as in a cell model STHdhQ^111/111^, precluding the possibility of secondary effects. Expectedly, overexpression of mutant Htt exon1 also increases Grb2 protein levels in Neuro2A cells. While investigating the mechanism of Grb2 upregulation in HD models, quite serendipitously it is seen that the transcription factor Foxd3 binds to the Grb2 upstream promoter region and activates its expression. Foxd3, which is upregulated in both R6/2 mice striatum and STHdhQ^111/111^ cells, hence upregulates Grb2 expression. The phosphorylation of downstream effectors of Grb2 in MAPK signaling pathway are found to be downregulated in STHdhQ^111/111^ cells. It is reported that downregulation of ERK1/2 phosphorylation increases the levels of Foxd3 [33]. This observation suggests the existence of a feedback loop in the regulation of Grb2, Foxd3 and ERK1/2 phosphorylation.

In an attempt to mimic a similar condition by overexpressing Grb2 (fusion product Grb2-Dsred) along with mutant Htt exon1 (as 145QHttex1GFP) in Neuro2A cells, Grb2 is found to reduce the number of cells having Htt aggregates as evident from the increase in mobility of Htt in presence of Grb2. Grb2 is known to activate Ras and subsequent effectors of MAPK signaling pathway [6]. Grb2 is also known to be involved in coated pit formation and EGF receptor internalization and endocytosis [34]. For the first time, we could demonstrate with clear evidences that Grb2 can have a chaperone like activity inside the cell. Grb2 is located inside endocytic vesicles and Htt exon1, when overexpressed, is found to colocalize with these Grb2 containing vesicles in Neuro2A cells. Endogenous Htt is found to colocalize with Grb2 in STHdhQ^111/111^ cells whereas no such colocalization is observed in the control cells. Earlier the Grb2-Htt interaction was reported to be regulated by EGFR activation [5]. We found that Htt can be pulled down by Grb2 only in case of STHdhQ^111/111^ cells, indicating that the association of Grb2 is directly correlated with the mutant form of Htt independent of EGFR activation. This polyQ length dependence of Grb2 interaction with Htt might have clinical implications.

Based on the observation that Grb2 is upregulated in STHdhQ^111/111^ cells, it is expected to activate Ras and enhance phosphorylation of MAPK effecter ERK1/2. On the contrary, phospho ERK1/2 level is found to be downregulated in STHdhQ^111/111^ cells. We report that the excess load of Grb2 in STHdhQ^111/111^ cells is redirected towards autophagic removal of mutant Htt and hence phospho ERK1/2 level is diminished instead. STHdhQ^111/111^ cells show upregulation of autophagy related marker proteins LC3 and Beclin1 and experiments with lysosomal inhibitor further validates this. In the same system LC3 is found to be colocalized with Htt and Grb2 complex. At this context a novel function of Grb2 as a ‘scavenger’ molecule may not be ruled out.

This involvement of Grb2 in the clearance of the toxic load of Htt is confirmed by knocking down Grb2 in STHdhQ^111/111^ cell which show downregulation of LC3 and delay in fusion of LC3 containing vesicles to lysosomes. Involvement of Grb2 in fusion of autophagic vesicles to lysosomes is also intriguing. The two novel functions of Grb2, like a chaperone and a scavenger protein, to specifically clear the toxic effect and load of mutant Htt, is of enormous importance in the context of HD pathology since the levels of endogenous Grb2 is naturally elevated in HD models. These emerging roles of Grb2 can be viewed as a natural protective mechanism of the cell to combat the disease.

So, according to our findings Grb2 preferentially interacts with mutant Htt in STHdhQ^111/111^ cells and this interaction acts as a competitor of Grb2-SOS1 interaction. As a result MAPK signaling is downregulated in these cells, as evident from lower phospho ERK1/2 levels in STHdhQ^111/111^. This in turn activates the Foxd3 levels in the cell which regulates the Grb2 levels. Upregulated Grb2 helps Htt clearance via autophagy-lysosomal pathway.

## Supporting Information

File S1 **Details of experimental procedures.**
(PDF)

File S2 **Supporting Figures (Figure S1- Figure S6) and Tables (Table S1 – Table S3).** Table S1, Table showing primer sequences used for Realtime PCR experiments and molecular cloning. Table S2, List of Transcription Factors From MATCH output with known expressions in HD. Table S3, List of Transcription Factors with unknown expressions in HD. Figure S1, Representative confocal image of neuro2a cell, co-transfected with Grb2 dsred and 145QHttexon1 and immunostained with Rab7 antibody and nuclear stained with DAPI. Figure S2, Bar diagram of the mean optical densities of bands obtained in three independent filter retardation assay (n=3) with neuro2A cells transfected with Grb2-Dsred; 145Qhttex1 GFP and Grb2-Dsred; 145Q httex1 GFP; and 145Q Httex1 and Dsred. In all cases total input loads were 20μg and 40μg. Figure S3, MTT assay result of three independent (n=3) experiments of STHdhQ^7/7^ and STHdhQ^111/111^ and STHdhQ^111/111^ Grb2 si cells and transfected with Grb2-dsred and Dsred respectively. Figure S4, Representative confocal images showing endogenous distribution of Htt, Grb2 and LC3 in STHdhQ^7/7^ and STHdhQ^111/111^ cells. Figure S5, A. ICQ analysis of images in B (n=20) B. Pearson’s correlation coefficient of images in B (n=20) of Fig.6. Figure S6, E. Representative confocal images of endogenous Htt expression in STHdhQ^7/7^ and STHdhQ^111/111^ cells transfected with DsredC1 empty vector and Grb2 dsred. F. Endogenous Htt expression in STHdhQ^111/111^ Grb2 si cells , STHdhQ^111/111^Grb2 si cells transfected with Grb2 dsred and Dsred C1 vector.
(PDF)
