# Supplementary material for: Grb2 Is Regulated by Foxd3 and Has Roles in Preventing Accumulation and Aggregation of Mutant Huntingtin
Source: PLoS One. 2013 Oct 8;8(10):e76792. doi: 10.1371/journal.pone.0076792 (PMC3792889; doi:10.1371/journal.pone.0076792)
Supplement: File S1 — Details of experimental procedures. (PDF) [file pone.0076792.s001.pdf]

Supplementary Material :Experimental Procedure Details:

R6/2 Mice:

All animal experiments were performed according to the protocol approved by the Institutional Animal Ethics Committee of National Brain Research Centre, Manesar. Animals had free access to pelleted diet and water *ad libitum.* All efforts were made to minimize animal suffering.

Plasmid Constructs:

Grb2 Dsred and Grb2 si, clones have been described previously[1]. Grb2-pET28A clone was kindly provided by Dr. Mithu Raychaudhuri. Htt exon1 plasmids 23Q, and 145Q were kindly provided by Dr. Patrick Lajoie [2], Albert Einstein College of Medicine, Yeshiva university, NY, USA. The pDest-mCherry-EGFP-LC3B plasmid [3] was kindly provided by Prof. Terje Johansen,University of Tromso . Hsp70-GFP clone was kindly provided by Mrs. Kamalika Roychoudhury, Saha Institute of Nuclear Physics, India.

Antibodies and Chemicals:

Hygromycin, Insulin from bovine pancreas, Hsp70 were obtained from Sigma Chemicals (MO,USA). The anti-mouse and anti-rabbit secondary antibodies conjugated with horseradish peroxidase were purchased from Bangalore Genei (India). Anti-Grb2(clone Y237, ab23037), anti-Rab7(ab50533), anti ERK1/2 (ab54230), anti FOXD3(ab67758), anti β-actin (ab6375) antibodies were purchased from abcam,USA , anti polyQ antibody was purchased from Chemicon,USA, anti Htt (#5656), anti Phospho p42/44 (#9101) were purchased from Cell Signaling Technology, USA , anti LC3 (NB100-2220), anti ATG5 (NB110-53818) and anti Beclin1 (NB500-249) antibodies were purchased from Novus Biologicals, USA. Alexa Fluor 633 F(ab')2 fragment of goat anti-rabbit IgG (H+L) (A21072), Alexa Fluor 546 F(ab')2 fragment of goat anti-rabbit IgG (H+L) (A11071) Alexa Fluor® 488 F(ab')2 fragment of goat anti-rabbit

IgG (H+L) (A11070). Alexa Fluor 546 F(ab')2 fragment of goat anti-mouse IgG (H+L) (A11018), Alexa Fluor® 633 F(ab')2 fragment of goat anti-mouse IgG (H+L) (A21053), Alexa Fluor® 488 F(ab')2 fragment of goat anti-mouse IgG (H+L) (A11017.) were purchased from Invitrogen, USA.

RNA Isolation from Mouse Tissue Samples:

In brief, isolation method for RNA from mouse tissue samples consists of the following steps: Rehydration: after paraffin solubilization, a rehydration step was introduced where the supernatants from the previous step were carefully removed and the pellets were successively washed with 1 ml of absolute ethanol and 1 ml of 95% ethanol in DEPC water. After each step the tissue was collected by centrifugation at 10,000 g for 10 min. Protein digestion: After the final wash, alcohol was aspirated and the tissue pellets were air dried in a thermoblock at 37°C and re-suspended in 500 ml of digestion buffer (10 mM NaCl, 500 mM Tris, pH 7.6, 20 mM EDTA and 1% SDS). To obtain purified RNA, tissue proteins were removed by adding 500 mg/ml of the proteolytic enzyme proteinase K. The sections were then incubated at 45°C for 16 hours. Prior to RNA purification, proteinase K was inactivated at 100°C for 7 min in order to nullify its effects on PCR. RNA extraction: Total RNA was then extracted from these tissue sections by using Trizol reagent and following manufacturer’s protocol. Concentrations of total RNA was measured and total RNA was used to measure expression levels of genes Grb2 , FOXD3 and beta-actin after making cDNA.

Protein Extraction and Western Blot Analysis:

PBS-washed pellets from cell lines were lysed on ice in lysis buffer (50 mM Tris·HCl, pH 7.5,150mM NaCl,15 mM EDTA, 0.5% Triton X-100) for 30 min in presence of Complete

protease inhibitor (Roche Diagnostics) and centrifuged at 13,000rpm for 15mins. Protein concentration was determined by Bradford protein estimation assay.

Proteins were separated on SDS–polyacrylamide gels and transferred onto PVDF membranes (Millipore Corporation), which were blocked by incubation in 5% dried milk in TBST (50 mM Tris-HCl, 150 mM NaCl, pH 7.5 containing 0.05% Tween 20). Membranes were probed with primary antibodies against specific proteins were then added to the blots. Immuno-reactive bands were detected with enhanced chemiluminescence reagent (Super Signal West Pico Substrate; Pierce) and signals were visualized by exposing the membranes to ECL Hyperfilm (Amersham Biosciences).

Quantification of western blots was carried out using Quantity One software of Bio-Rad. At least three separate experiments were analyzed and band intensities were normalized to loading control (beta actin).

Immunocytochemistry

Cells were grown on poly-L-lysine-coated glass coverslips. Cells were washed in PBS and fixed in 0.1 M PBS containing 4% para-formaldehyde for 30 min at room temperature and further permeabilized with 0.25% (v/v) Triton X-100 in PBS. After blocking in 1% (w/v) bovine serum albumin, the fixed materials were incubated for 2 h at room temperature with the primary antibody in PBS containing 1% (w/v) bovine serum albumin and 0.25% Triton X-100. After washing, alexa tagged secondary antibody was used. Coverslips were mounted on slides and images were acquired with confocal microscope by using the appropriate laser lines.

Chromatin Immunoprecipitation:

In brief, Cells were crosslinked with 1.1% formaldehyde for 10 minutes at room temperature. This crosslinking reaction was stopped using 125mM Glycine. Cells were washed with ice cold

PBS and harvested at 300g for 3 min at 4°C. Cytosol was extracted using buffer C (20mM $MgCl_2$, 0.2mM EDTA and 1mM PMSF). Nuclei were harvested at 13,000g for 10 min at 4°C and pellet was resuspended in breaking buffer (50mM Tris-HCl pH8.0, 1mM EDTA, 150mM NaCl, 1% SDS and 2% Triton-X-100) was added to the nuclear extract. Immunoprecipitation reaction was carried out using anti-FOXD3 antibody followed by addition of protein A/G beads. The immunoprecipitated complex was washed followed by de-crosslinking, phenol **chloroform** extraction and ethanol precipitation of the DNA. PCR amplification of the eluted DNA was carried out using sequence specific primers for Grb2 upstream sequence. The primer sequence are given in supplementary Table S1.

Luciferase Assay:

Briefly, cells grown in 35-mm plates were transfected with 500 ng of pGL3 construct (empty pGL3 vector or Luc-Grb2ups construct). Twenty-four hour after transfection, the luciferase assay was carried out using the luciferase reporter assay system (Promega) according to the manufacturer’s protocols and detected by a Sirius Luminometer (Berthold Detection Systems). Three microgram of protein was used for each assay. Transfection efficiency was normalized by co-transfecting with eGFP-C1 (clontech) and measuring the fluorescence at 510nm (Fluromax-3, Jobin Yvon Horiba, USA).

MTT Assay:

10^4Cells were cultured in 24 well plates, 48hrs post transfection, cell survivility was assayed using (3-(4,5-Dimethylthiazol-2-yl)-2,5-diphenyltetrazolium bromide (MTT) dye (Cat no. 209410, SRL, India). MTT at conc. 100microgram/ml was added to plates and allowed to incubate for 4 hours. Following which the culture medium was discarded and cells were lysed in

acidic isopropanol. Reading was taken at 570nm for MTT and for background subtraction at 650nm using spectrophotometer (Smartspec Plus, Biorad, USA).

In-vitro Chaperone Assay:

Insulin was dissolved in a minimum volume of 20 mM NaOH and then diluted to the required concentration (0.3 mg/ml) in 100 mM phosphate buffer, pH 7.0. The reduction of insulin was initiated by adding 20μl of 1M DTT to 1 ml in absence and presence of 0.3 mg.ml Grb2 , BSA and Hsp70, the latter being used as the positive control. The aggregation of the insulin B chain was measured as a function of time at 25°C by monitoring the apparent absorbance (scattering) at 360nm.

Reference

1. Raychaudhuri M, Mukhopadhyay D (2010) Grb2-mediated alteration in the trafficking of AbetaPP: insights from Grb2-AICD interaction. J Alzheimers Dis 20: 275-292.

2. Lajoie P, Snapp EL (2010) Formation and toxicity of soluble polyglutamine oligomers in living cells. PLoS One 5: e15245.

3. Pankiv S, Clausen TH, Lamark T, Brech A, Bruun JA, et al. (2007) p62/SQSTM1 binds directly to Atg8/LC3 to facilitate degradation of ubiquitinated protein aggregates by autophagy. J Biol Chem 282: 24131-24145.
