# Supplementary material for: Grb2 Is Regulated by Foxd3 and Has Roles in Preventing Accumulation and Aggregation of Mutant Huntingtin
Source: PLoS One. 2013 Oct 8;8(10):e76792. doi: 10.1371/journal.pone.0076792 (PMC3792889; doi:10.1371/journal.pone.0076792)
Supplement: File S2 — Supporting Figures (Figure S1- Figure S6) and Tables (Table S1 – Table S3). Table S1, Table showing primer sequences used for Realtime PCR experiments and molecular cloning. Table S2, List of Transcription Factors From MATCH output with known expressions in HD. Table S3, List of Transcription Factors with unknown expressions in HD. Figure S1, Representative confocal image of neuro2a cell, co-transfected with Grb2 dsred and 145QHttexon1 and immunostained with Rab7 antibody and nuclear stained with DAPI. Figure S2, Bar diagram of the mean optical densities of bands obtained in three independent filter retardation assay (n=3) with neuro2A cells transfected with Grb2-Dsred; 145Qhttex1 GFP and Grb2-Dsred; 145Q httex1 GFP; and 145Q Httex1 and Dsred. In all cases total input loads were 20μg and 40μg. Figure S3, MTT assay result of three independent (n=3) experiments of STHdhQ7/7 and STHdhQ111/111 and STHdhQ111/111 Grb2 si cells and transfected with Grb2-dsred and Dsred respectively. Figure S4, Representative confocal images showing endogenous distribution of Htt, Grb2 and LC3 in STHdhQ7/7 and STHdhQ111/111 cells. Figure S5, A. ICQ analysis of images in B (n=20) B. Pearson’s correlation coefficient of images in B (n=20) of Fig.6. Figure S6, E. Representative confocal images of endogenous Htt expression in STHdhQ7/7 and STHdhQ111/111 cells transfected with DsredC1 empty vector and Grb2 dsred. F. Endogenous Htt expression in STHdhQ111/111 Grb2 si cells , STHdhQ111/111Grb2 si cells transfected with Grb2 dsred and Dsred C1 vector. (PDF) [file pone.0076792.s002.pdf]

SUPPLEMENTARY MATERIALS :TABLES AND FIGURES

Supplementary Table S1

Table showing primer sequences used for Realtime PCR experiments and molecular cloning

Primer Name	Sequence
Grb2_ups_For	5’-CCG CTC GAG TCT TCT GCT GCT TCT CTC GAT-3’
Grb2_ups_Rev	5’-CCC AAG CTT TCA CGT CCT TTC TAC CAA AAC A-3’
Grb2_RT_1	5’-AAA TGC TCA GCA AAC AGC GG-3’
Grb2_RT_2	5’-TGA AGT GCT GCA CAT CAT TTC C-3’
FOXD3_RT_1	5’- TCT GCG AGT TCA TCA GCA ACG-3’
FOXD3_RT_2	5’- TTG ACG AAG CAG TCG TTG AGC-3’
Beta actin_RT-1	5’-TCC TGT GGC ATC CAG GAA ACT-3’
Beta actin_RT_2	5’- GAA GCA TTT GCG GTG GAC-3’

Supplementary Table S2

List of Transcription Factors From MATCH output with known expressions in HD

Transcription Factor	Binding site position	Core Match	Matrix Match	Expression in HD	Regulation
HNF-3beta	225	1.000	0.975	Upregulated	Positive/ negative
FOXD3	226	1.000	0.963	Upregulated	Positive/ negative
Pax-6	2908<br>9271	0.792<br>1.000	0.804<br>0.833	Up/downreulated	negative
Pax-4	4676<br>8702	0.979<br>1.000	0.836<br>0.843	downregulated	negative
Elk-1	5641	1.000	0.922	downregulated	positive
Evi-1	7620	0.842	0.862	upregulated	negative

Supplimentary Table S3

List of Transcription Factors with unknown expressions in HD

Transcription Factor	Binding site position	Core Match	Matrix Match
v-Maf	1152	1.000	0.921
v-Myb	1682	1.000	0.966
	1802	1.000	0.971
	7269	1.000	0.971
HNF-1	2734	0.794	0.844
CCAAT box	2752	1.000	0.981
Oct-1	2867	1.000	0.940
	4655	0.909	0.893
	8435	0.992	0.990
NF-Y	2988	1.000	0.992
AP-1	3052	1.000	0.991
	4689	1.000	0.982
HNF-1	6364	0.942	0.868
COMP-1	5825	1.000	0.814
	6942	0.822	0.825
GATA-1	7566	0.993	0.991
Hand1	7799	1.000	0.961
USF	8117	1.000	1.000

<u>Reference for Table S2:</u>

<u>[1]</u> Hodges, A., Strand, A. D., Aragaki, A. K., Kuhn, A., Sengstag, T., Hughes, G., Elliston, L. A., Hartog, C., Goldstein, D. R., Thu, D., Hollingsworth, Z. R., Collin, F., Synek, B., Holmans, P. A., Young, A. B., Wexler, N. S., Delorenzi, M., Kooperberg, C., Augood, S. J., Faull, R. L., Olson, J. M., Jones, L., and Luthi-Carter, R. (2006) *Hum Mol Genet* **15**, 965-977

<u>[2]</u> Lee, J. M., Ivanova, E. V., Seong, I. S., Cashorali, T., Kohane, I., Gusella, J. F., and

MacDonald, M. E. (2007) *PLoS Genet* **3**, e135

[3] Gene expression atlas http://www.ebi.ac.uk/gxa/

[4] Transfac Match http://www.gene-regulation.com/pub/programs.html

Supplementary Figure legends:

Fig S1: Representative confocal image of neuro2a cell, co-transfected with Grb2 dsred and 145QHttexon1 and immunostained with Rab7 antibody and nuclear stained with DAPI.

Fig S2: Bar diagram of the mean optical densities of bands obtained in three independent filter retardation assay (n=3) with neuro2A cells transfected with Grb2-Dsred; 145Qhttex1 GFP and Grb2-Dsred; 145Q httex1 GFP; and 145Q Httex1 and Dsred. In all cases total input loads were 20μg and 40μg.

Fig S3: MTT assay result of three independent (n=3) experiments of STHdhQ7/7 and STHdhQ111/111 and STHdhQ111/111 Grb2 si cells and transfected with Grb2-dsred and Dsred respectively.

Fig S4: Representative confocal images showing endogenous distribution of Htt, Grb2 and LC3 in STHdhQ7/7 and STHdhQ111/111 cells.

Fig S5: A. ICQ analysis of images in B (n=20) B. Pearson's correlation coefficient of images in B (n=20) of Fig.6.

Fig S6: E. Representative confocal images of endogenous Htt expression in STHdhQ7/7 and STHdhQ111/111 cells transfected with DsredC1 empty vector and Grb2 dsred. F. Endogenous Htt expression in STHdhQ111/111 Grb2 si cells , STHdhQ111/111Grb2 si cells transfected with Grb2 dsred and Dsred C1 vector.

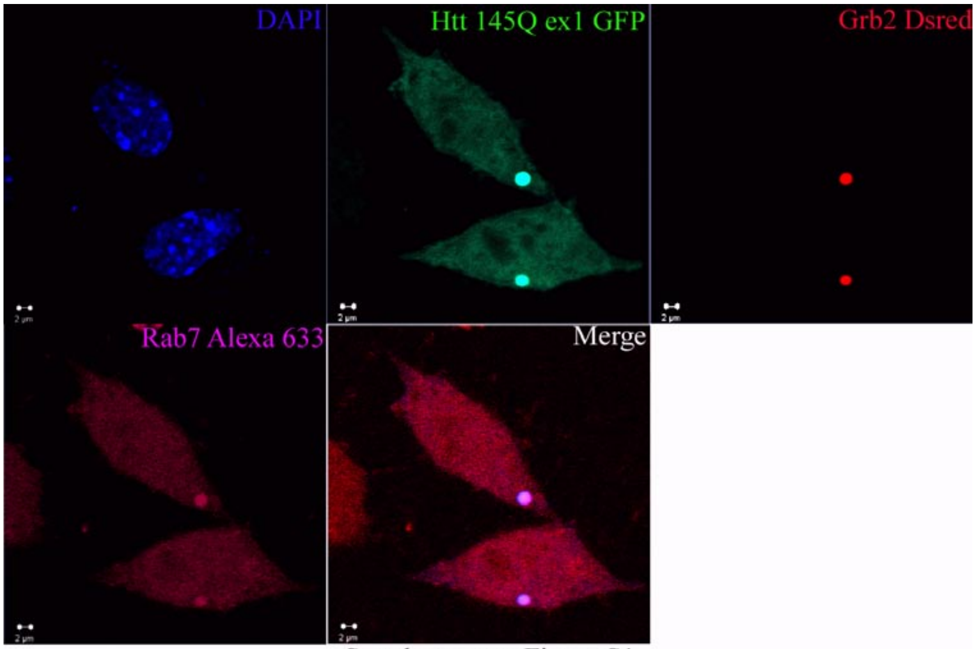

Supplementary Figure S1

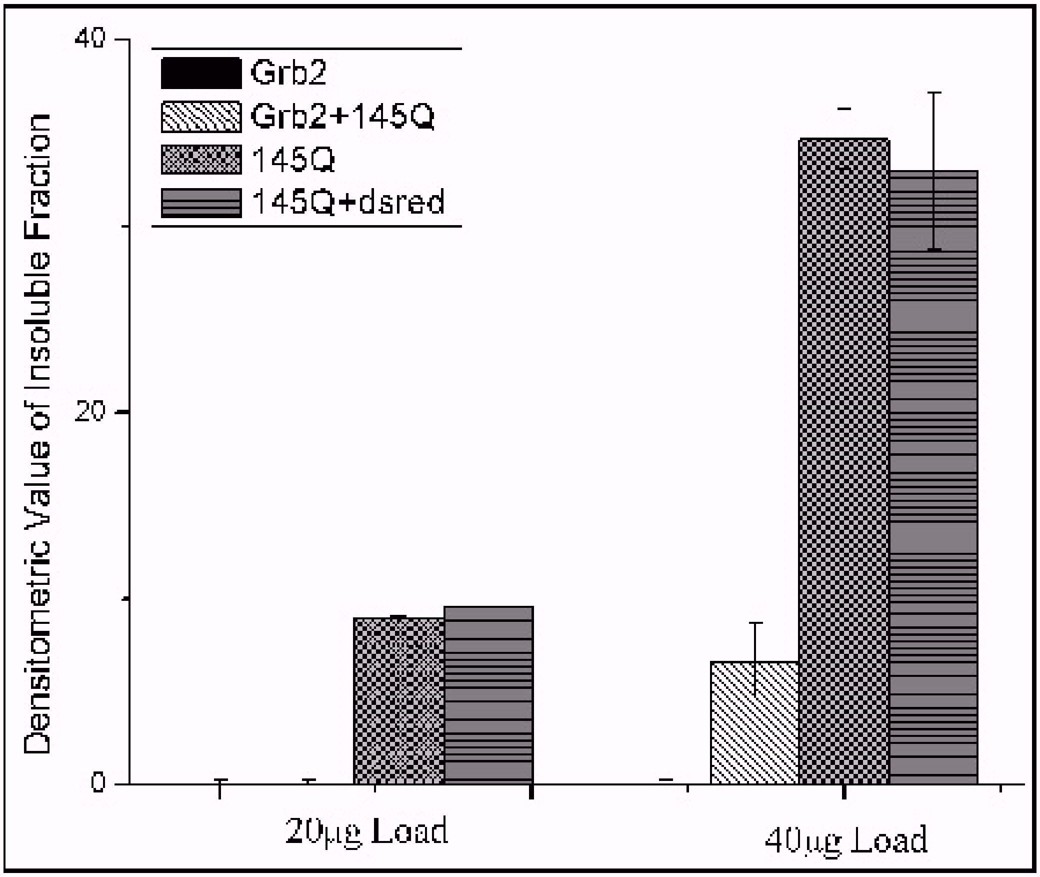

Supplementary Figure S2

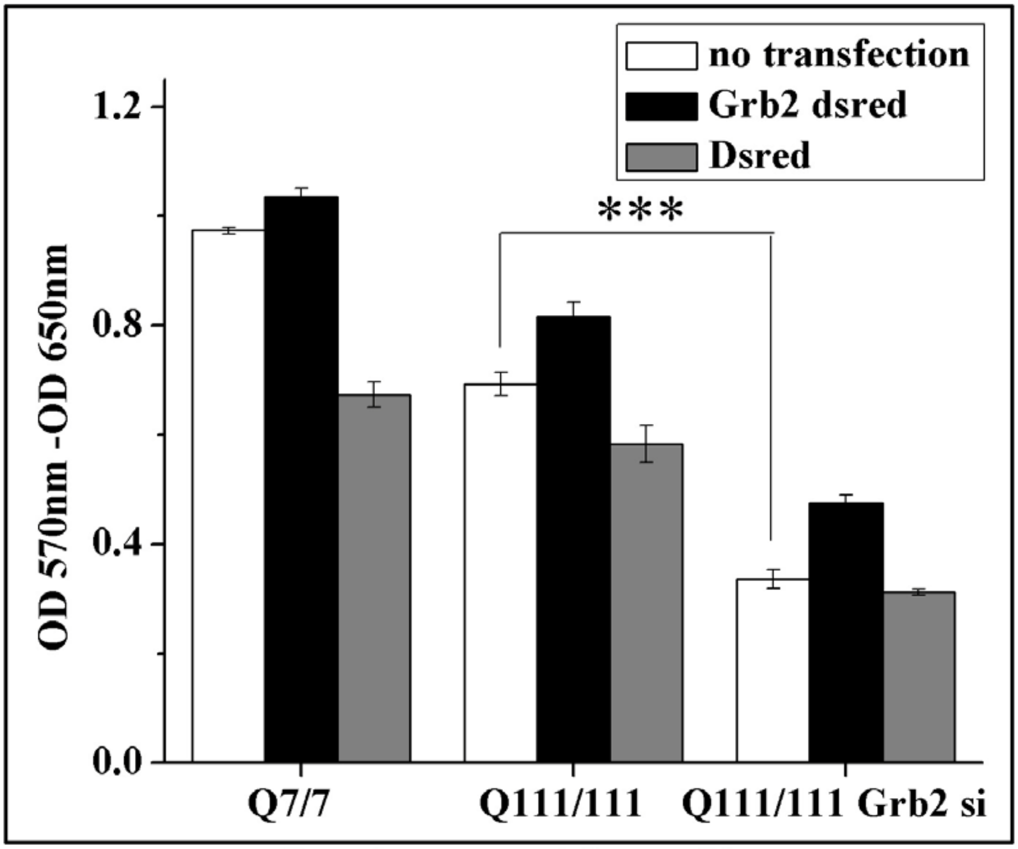

Supplementary Figure S3

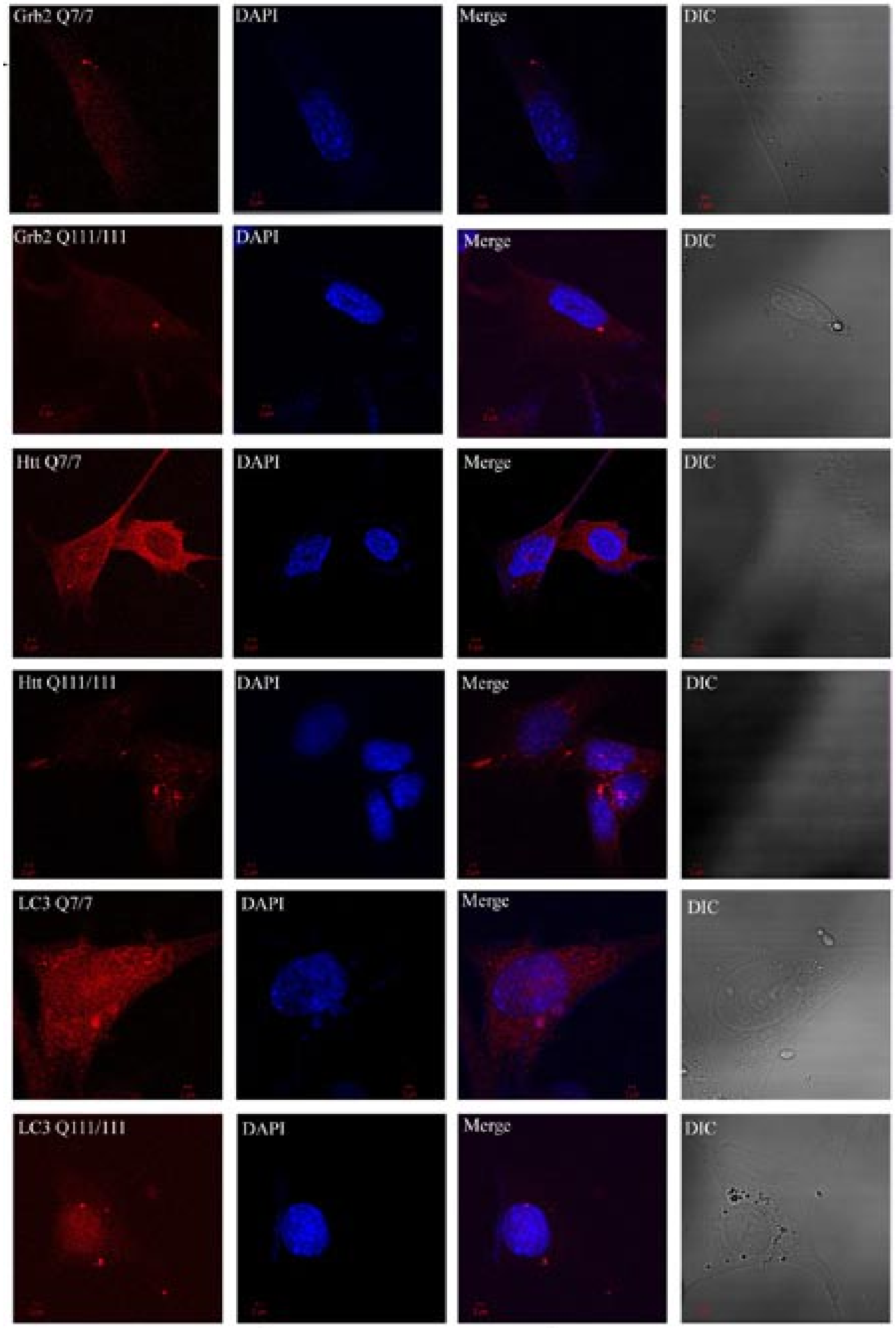

Supplementary Figure S4

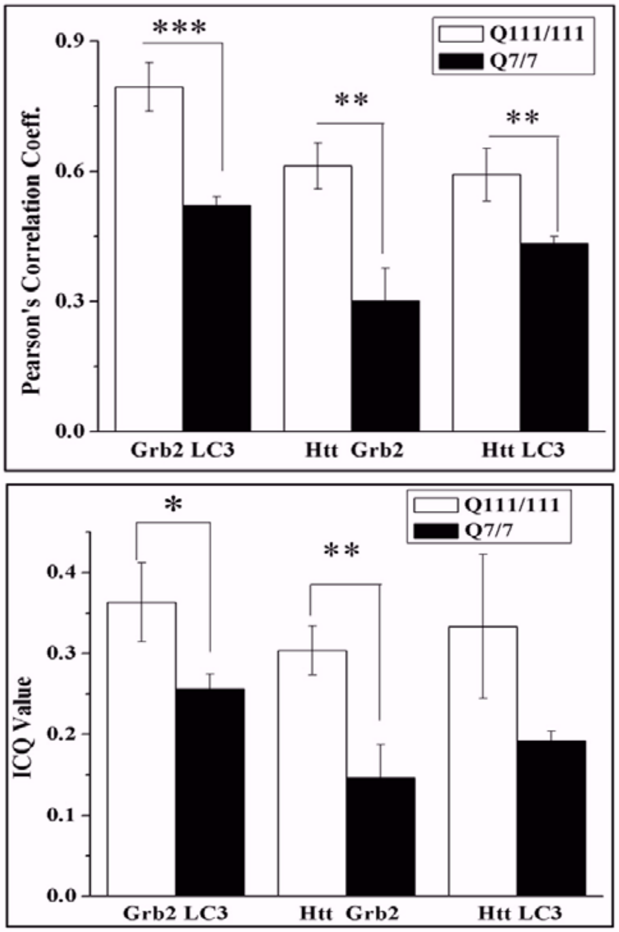

Supplementary Figure S5

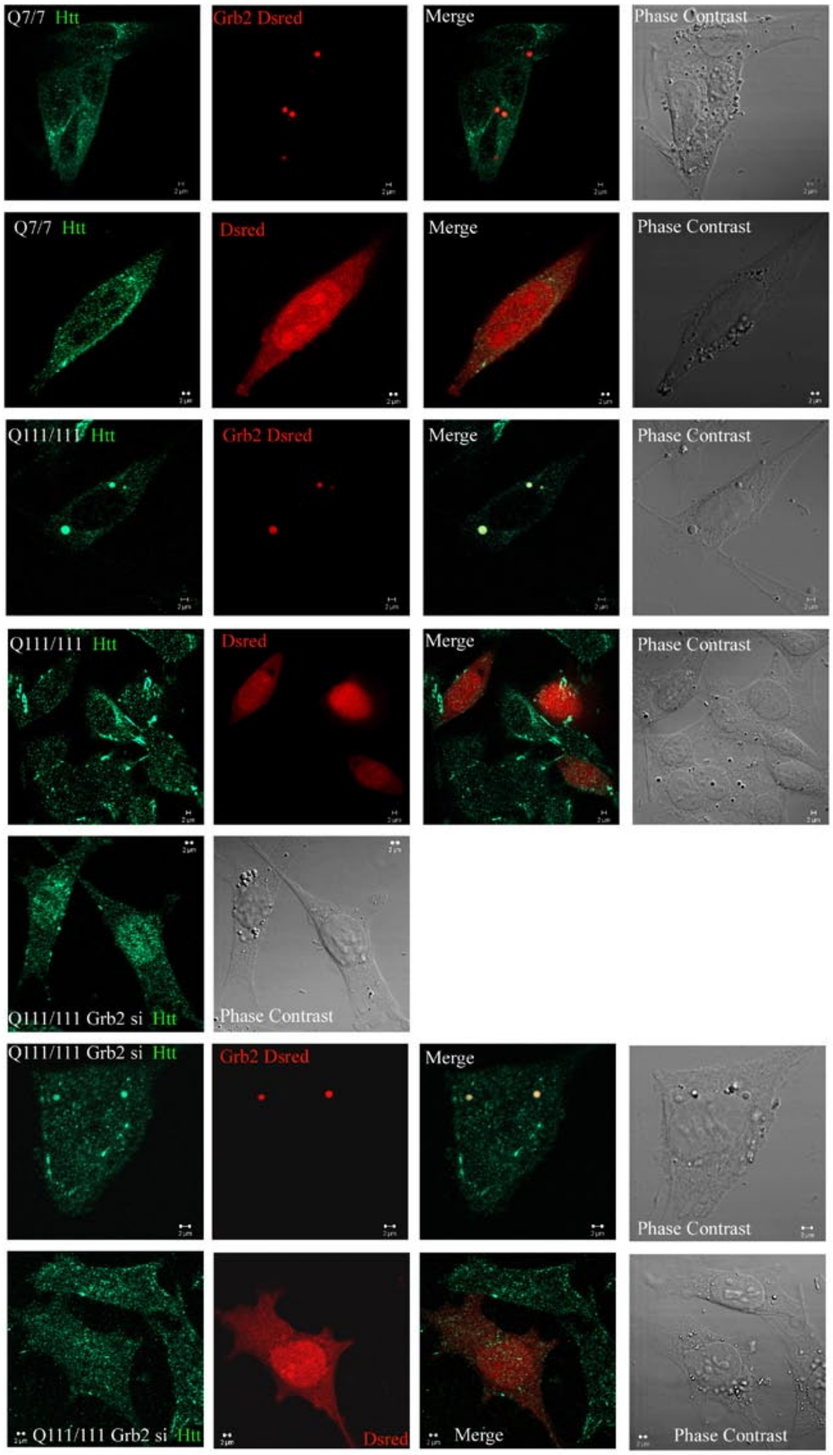

Supplementary Figure S6
